# Interleukin-1**β** Accelerates the Onset of Stroke in Stroke-Prone Spontaneously Hypertensive Rats

**DOI:** 10.1155/2012/701976

**Published:** 2012-12-27

**Authors:** Tsuyoshi Chiba, Tatsuki Itoh, Masaki Tabuchi, Toru Nakazawa, Takao Satou

**Affiliations:** ^1^Information Center, National Institute of Health and Nutrition, 1-23-1 Toyama, Shinjuku-ku, Tokyo 162-8636, Japan; ^2^Department of Pathology, Kinki University School of Medicine, 377-2 Ohno-higashi, Osaka-sayama, Osaka 589-8511, Japan; ^3^Department of Biochemistry, Kinki University School of Medicine, 377-2 Ohno-higashi, Osaka-sayama, Osaka 589-8511, Japan; ^4^First Institute of New Drug Discovery, Otsuka Pharmaceutical Co., Ltd., Tokushima 771-0192, Japan; ^5^Division of Hospital Pathology, Hospital of Kinki University School of Medicine, 377-2 Ohno-higashi, Osaka-sayama, Osaka 589-8511, Japan

## Abstract

High blood levels of inflammatory biomarkers and immune cells in stroke lesions have been recognized as results of stroke. However, recent studies have suggested that inflammation occurs prior to stroke onset. In this study, we aimed to clarify the role of inflammation in stroke onset among stroke-prone spontaneously hypertensive rats (SHRSP). At 4 weeks of age (before stroke onset), the plasma level of IL-1**β** was significantly higher in SHRSP (153.0 ± 49.7 pg/ml) than in Wistar Kyoto rats (WKY) (7.7 ± 3.4 pg/ml, *P* < 0.001 versus SHRSP) or spontaneously hypertensive rats (SHR) (28.0 ± 9.1 pg/ml, *P* < 0.001 versus SHRSP) (*n* = 6 per strain). Stimulated IL-1**β** signal was also observed in cerebrovascular endothelial cells of SHRSP. Gene expressions of IL-1**β**, IL-1 receptors, caspase-1, and downstream genes (MCP-1 and ICAM-1), which associated with immune cell recruitment, were significantly greater in SHRSP than in WKY or SHR, coincident with greater NF**κ**B protein levels in SHRSP compared to WKY or SHR. In addition, continuous administration of IL-1**β** (2 **μ**g/day) using an osmotic pump slightly increased the incidence of stroke in SHR (*P* = 0.046) and significantly accelerated the onset of stroke in SHRSP (*P* = 0.006) compared to each control (*n* = 10 per group). These results suggest that a stimulated IL-1**β** signal might be a cause of stroke onset when concomitant with severe hypertension.

## 1. Introduction

Inflammatory processes play a central role in the pathogenesis of vascular diseases and their complications. In the clinical setting, elevated plasma levels of inflammatory cytokines, C-reactive protein (CRP), and chemokines are associated with future cardiovascular risk [1]. Further, serum levels of intercellular adhesion molecule-1 (ICAM-1) and monocyte chemoattractant protein-1 (MCP-1) are high in patients with ischemic stroke and myocardial infarction [2, 3], which might be interpreted as a stroke-induced increase in inflammatory events. However, plasma levels of ICAM-1 and MCP-1 are also elevated in patients with essential hypertension in the absence of other diseases [4]. Thus, inflammation is a risk factor for stroke in hypertensive subjects, but whether it is a cause of stroke remains unclear [5].

Treatment with drugs, which have anti-inflammatory properties, can prevent stroke in humans and in animal models. In healthy persons without hyperlipidemia but with elevated high-sensitivity CRP level, rosuvastatin, which lowers high-sensitivity CRP as well as cholesterol level, reduced the incidence of stroke and myocardial infarction by 50% relative to placebo [6]. Stroke-prone spontaneously hypertensive rats (SHRSP), a unique genetic model of hemorrhagic stroke [7], have previously been used to examine the contributions of inflammation to stroke. In SHRSP fed a high-salt diet, rosuvastatin treatment significantly delayed the onset of stroke and attenuated the transcription of inflammatory biomarkers (MCP-1, transforming growth factor-*β*1, interleukin (IL)-1*β*, and tumor necrosis factor-*α* (TNF-*α*)) [8]. Further, pioglitazone delayed the onset of stroke by improving vascular endothelial dysfunction, inhibiting brain inflammation, and reducing oxidative stress [9], and low-dose acetylsalicylic acid (aspirin) also delayed the onset of stroke in SHRSP via the suppression of inflammation [10]. Frequent aspirin use might also confer a protective effect for risk of hemorrhagic stroke in humans [11]. In addition to these anti-inflammatory drugs, we previously reported that dietary restriction suppressed systemic and local inflammation and significantly delayed the onset of stroke and extended lifespan in SHRSP [12]. We also reported that plasma IL-1*β* level was high when compared with IL-6 or TNF-*α* level in SHRSP, and that dietary restriction significantly decreased plasma IL-1*β* level. It seems that dietary restriction delayed the onset of stroke via a decrease in plasma IL-1*β* level.

We found that plasma level of IL-1*β*, but not IL-6 or TNF-*α*, was significantly greater in SHRSP than in WKY or SHR in this report. IL-1*β* increased the expression of adhesion molecules (ICAM-1 and vascular cell adhesion molecule-1) and MCP-1 in cerebrovascular endothelial cells (CVECs) [12], which stimulated monocytes/macrophages infiltration into brain and induced blood-brain barrier damages [13, 14]. These mechanisms might be underlying in the stroke onset. In addition to animal studies, IL-1 signal is focused as the therapeutic target for stroke in humans [15]. Therefore, the goal of the present study was to clarify the role of inflammation, especially IL-1*β*, in the onset of stroke in SHRSP.

## 2. Materials and Methods

### 2.1. Animals

Wistar Kyoto rats/Izm (WKY), spontaneously hypertensive rats/Izm (SHR), and SHRSP/Izm (male) were purchased from Japan SLC Inc. (Shizuoka, Japan) and maintained under specific pathogen-free conditions in a temperature-controlled room (22 ± 2°C) with a 12-hour light/dark cycle. Each animal was kept in a separate steel cage. All animal experiments were conducted with the approval of the National Institute of Health and Nutrition Ethics Committee on Animal Research.

### 2.2. Food Intake, Body Weight, and Blood Pressure

 Food intake, body weight, and blood pressure were measured as described previously [16].

### 2.3. Plasma Cytokine Levels

WKY, SHR, and SHRSP (male, 4 weeks of age, *n* = 5 in each strain) were allowed to fast overnight. Blood was drawn from the abdominal aorta under anesthesia, and plasma was prepared rapidly. Plasma IL-1*β*, IL-6, and TNF-*α* levels were determined with the Quantikine rat IL-1*β* (the minimum detectable dose is 5 pg/mL), IL-6 (the minimum detectable dose is 14–36 pg/mL), or TNF-*α* (the minimum detectable dose is 5 pg/mL) Immunoassay kit, respectively, (R&D Systems, Inc., Minneapolis, MN, USA). Plasma CRP level was determined with the rat C-reactive protein ELISA test kit (the minimum detectable dose is 3.9 ng/mL) (Life Diagnostics, Inc., West Chester, PA, USA).

### 2.4. Cerebrovascular Endothelial Cells

WKY, SHR, and SHRSP (male, 4 weeks of age, *n* = 5 in each strain) were killed under anesthesia. Brains were removed, and CVECs were harvested as described previously [16]. CVECs were cultured with Endothelial Cell Basal Medium-2 (Lonza, Walkersville, MD, USA) and used within 10 passage numbers.

### 2.5. Quantitative Real-Time PCR

Total RNA was extracted from CVECs with TRIzol Reagent (Invitrogen, Carlsbad, CA, USA) and reverse transcribed with Omniscript RT kit (QIAGEN, GmbH, Hilden, Germany) and oligo (dT) primers. Reactions were performed in 96-well plates with SYBR Green PCR Master Mix and a 7500 Real-Time PCR System (Applied Biosystems, Foster City, CA, USA). Results are expressed as the copy number ratio of the target mRNA to *β*-actin mRNA. The following rat-specific primer pairs were used:

 IL-1*β* forward 5′-AGCAGCTTTCGACAGTGAGGAGAA-3′;
 reverse 5′-TCTCCACAGCCACAATGAGTGACA-3′;
 IL-1RI forward 5′-AATGCATGGCGGCACCATAATCTG-3′;
 reverse 5′-CCATCTTGGCGGGAACAAACCAAA-3′;
 IL-1RII forward 5′-AAGGTTCAGGGCACACATGTCCTA-3′;
 reverse 5′-ATTGGTTCCGTTCTCCGTGTGAGT-3′;
 Caspase-1 forward 5′-GAAAGAATTTGCTGCCTGCCCAGA-3′;
 reverse 5′-GCTTGTCTTTCAAGCTTGGGCACT-3′;
 MCP-1 forward 5′-TGCTGTCTCAGCCAGATGCAGTTA-3′;
 reverse 5′-TACAGCTTCTTTGGGACACCTGCT-3′;
 ICAM-1 forward 5′-TGCAGGTGAACTGCTCTTCCTCTT-3′;
 reverse 5′-AGCTTCCAGTTGTGTCCACTCGAT-3′;
 
*β*-actin forward 5′-TTGCTGACAGGATGCAGAAGGAGA-3′;
 reverse 5′-ACTCCTGCTTGCTGATCCACATCT-3′.

### 2.6. Western Blot Analysis

Whole protein from CVECs was extracted with PRO-PREP (iNtRON Biotechnology, Inc., Gyeonggi-do, Korea) according to the manufacturer's instructions. CVECs (90-mm dish) were lysed in 600 *μ*L PRO-PREP and incubated for 30 minutes at −20°C. Then samples were centrifuged (15,000 ×g, 5 minutes, 4°C), and the supernatant was collected as the whole protein fraction. Protein (10 *μ*g) separated by SDS-PAGE (12.5% gel) was electrophoretically transferred onto Immun-Blot PVDF Membrane (Bio-Rad Laboratories, Hercules, CA, USA) and was then immunoblotted with specific primary antibodies: NF*κ*B p50 (sc-7178), p52 (sc-298), p65 (sc-372), RelB (sc-226), c-Rel (sc-71), and I*κ*B-*α* (sc-1643) (1 : 200–1000 dilution, Santa Cruz Biotechnology, Inc., Santa Cruz, CA, USA). Peroxidase-conjugated anti-rabbit IgG or anti-mouse IgG (1 : 2000 dilution, GE Healthcare, Buckinghamshire, UK) was used as the secondary antibody. Bands were visualized by enhanced chemiluminescence system (GE Healthcare), and were analyzed with NIH image (National Institutes of Health, Bethesda, MD, USA).

### 2.7. IL-1*β* Administration

SHR or SHRSP (male, 10 weeks of age) were subcutaneously administrated recombinant rat IL-1*β* (2 *μ*g/day) (PeproTech Inc., Rocky Hill, NJ, USA) or vehicle (phosphate-buffered saline) using osmotic pumps (Alzet model 2ML4 (2.5 *μ*L/hr for 28 days); Alzet Corp, Cupertino, CA, USA) and were fed a normal chow diet (caloric composition: 20% protein, 10% fat, and 70% carbohydrate), which was purchased from Research Diet Inc. (New Brunswick, NJ, USA) ad libitum and given drinking water supplemented with 1% NaCl. SHRSP were kept until death by stroke, and SHR were killed under anesthesia after a 4-week-administration period. Each group consisted of 10 animals.

### 2.8. Onset of Stroke

Onset of stroke was assessed by the appearance of neurologic symptoms and physiological changes as described previously [16].

### 2.9. Histological Analysis

The brain was fixed with 10% buffered formalin solution, sliced into six coronal sections, embedded in paraffin, cut into 4-*μ*m-thick sections, and stained with hematoxylin-eosin. Hemorrhage area was defined as area with clustering blood cells. Infarction area was defined as area with necrosis, gliosis, and glial scar. Lesions of infarction, hemorrhage, or edema were classified into five degrees by the percentage of lesion area to whole area in each section: (−), no abnormality; (+), 0–10%; (++), 10–20%; (+++), 20–30%; (++++), 30–40%; (+++++), more than 40%.

### 2.10. Statistical Analysis

Data are presented as the mean ± SD. Statistical analysis was conducted using one-way ANOVA with a Bonferroni post hoc test for parametric data (Statview 5.0, Abacus Concepts, Piscataway, NJ, USA). Data from two groups were compared with the unpaired Student's *t*-test. For stroke onset and survival curves, a Kaplan-Meier curve was obtained, and the comparison of groups was performed using the Log-Rank test. A *P* value < 0.05 was considered statistically significant.

## 3. Results

### 3.1. Body Weight, Tissue Weight, and Blood Pressure of Each Strain

At 4 weeks of age, WKY were normotensive, SHR and SHRSP were hypertensive. SHR and SHRSP showed significantly higher blood pressure compared to WKY, whereas, there was no significant difference in blood pressure between SHR and SHRSP (Table 1). At 10 weeks of age, WKY were still normotensive, SHR were hypertensive, and SHRSP were severe hypertensive. SHRSP showed significantly higher blood pressure compared to WKY and SHR (Table 1). However, SHRSP did not show any stroke symptoms at this age. Body weight and the liver weight were significantly lower in SHRSP than in WKY or SHR at both 4 and 10 weeks of age. Weight of the epididymal adipose tissue was not different among the three rat strains at 4 weeks of age; however, it was significantly lower in SHRSP than in WKY or SHR at 10 weeks of age.

### 3.2. SHRSP Had Systemically and Locally Higher Inflammatory States Compared with WKY and SHR

**Figure 1:**
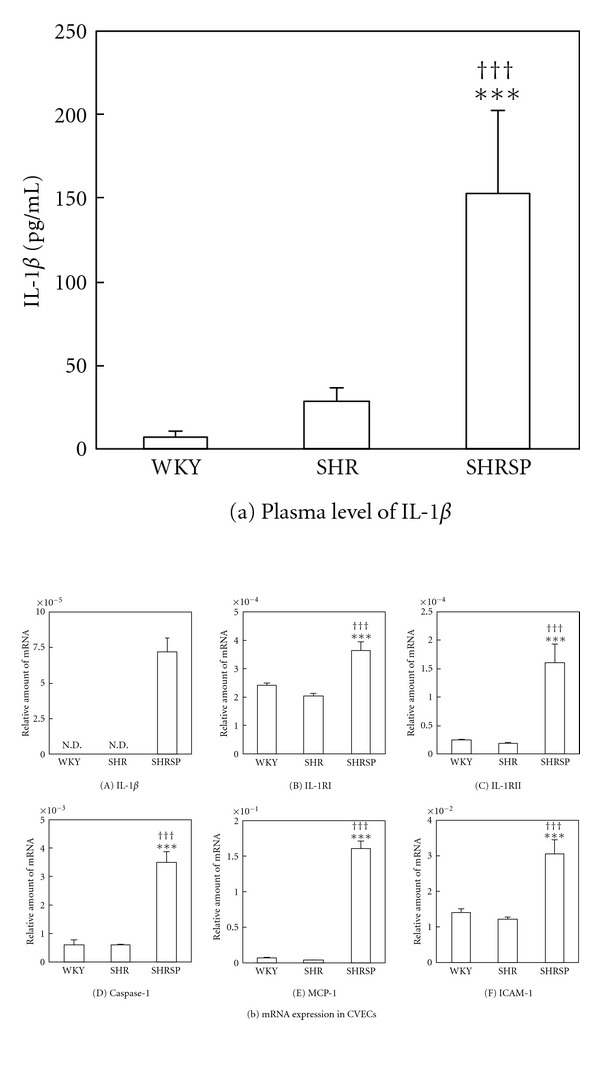
IL-1*β* level in plasma and IL-1*β* signal-related gene expression in CVECs from each strain. Plasma and CVECs were harvested from WKY, SHR, and SHRSP (male, 4 weeks of age). Total RNA was extracted for quantitative real time PCR. (a) IL-1*β* level in plasma and (b) IL-1*β* signal-related gene expression, such as (A) IL-1*β*, (B) IL-1RI, (C) IL-1RII, (D) caspase-1, (E) MCP-1, and (F) ICAM-1 in CVECs were measured as described in the methods section. Bars show SD, ***P < 0.001 versus WKY, ^†††^ *P* < 0.001 versus SHR. *n* = 5 in each strain. N.D.: not detectable.

At 4 weeks of age, plasma levels of inflammatory cytokines in WKY, SHR, and SHRSP were also measured. There was no significant difference in IL-6 and CRP levels among the three strains (data not shown), and TNF-*α* was undetectable in our assay system in all strains. On the other hand, IL-1*β* level was significantly higher in SHRSP than in WKY or SHR at 4 weeks of age (Figure 1(a)). These results indicated that SHRSP had a higher inflammatory state, especially IL-1*β*, compared with WKY or SHR, even though SHRSP were not obese compared with WKY or SHR (Table 1).

**Figure 2:**
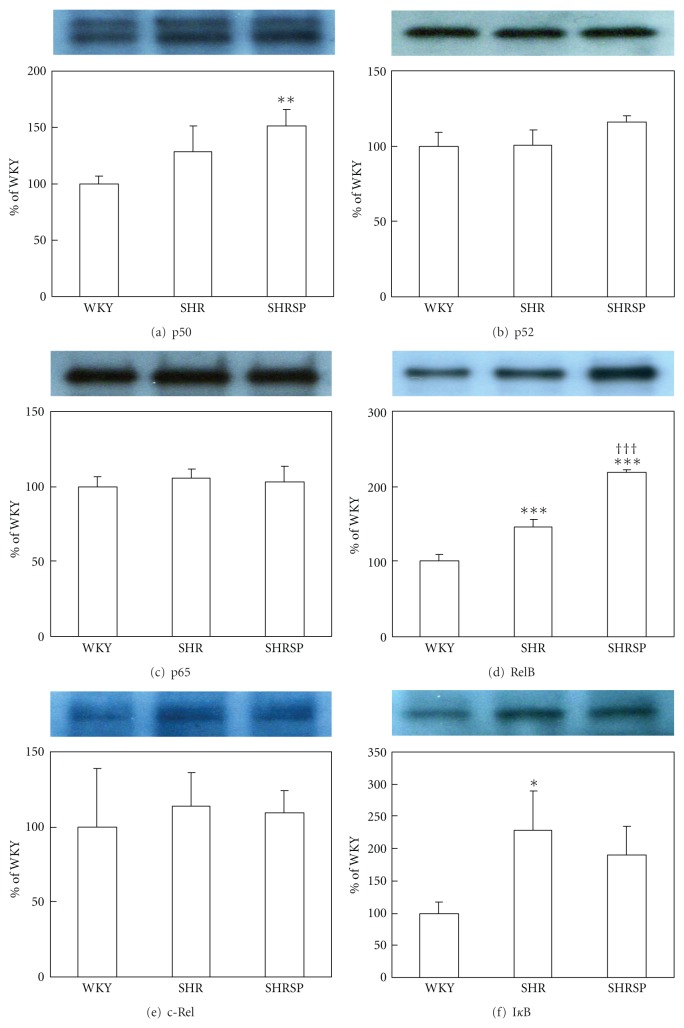
NF*κ*B protein levels in CVECs from each strain. CVECs were harvested from WKY, SHR, and SHRSP (male, 4 weeks of age). Whole cell protein was extracted for western blot analysis. Data are presented as mean ± SD of densitometric ratios (WKY was set as 100) of three independent experiments. *P < 0.05, **P < 0.01, ***P < 0.001 versus WKY, ^†††^ *P* < 0.001 versus SHR.

To address the role of IL-1*β* in stroke onset, IL-1*β* signal-related gene expression was measured in CVECs, the phenotype of which is important for stroke onset. IL-1*β* mRNA expression in CVECs could be detected in SHRSP, whereas it was undetectable in WKY and SHR (Figure 1(b)). In addition, mRNA expression of IL-1 receptors (IL-1RI and IL-1RII), IL-1*β* processing enzyme (caspase-1), and downstream genes of IL-1*β* signal (MCP-1 and ICAM-1) were also significantly greater in SHRSP than in WKY or SHR (Figure 1(b)). Furthermore, treatment with exogenous IL-1*β* (10 ng/mL) for 24 hours increased not only downstream genes (MCP-1 and ICAM-1), but also IL-1*β* mRNA expression in SHRSP CVECs through a positive feedback loop (data not shown). Protein levels of NF*κ*B, an IL-1*β* signal related transcription factor, were also determined in CVECs from each rat strain. p50 and RelB protein levels were significantly greater in SHRSP than in WKY and SHR (Figure 2). On the other hand, protein level of an endogenous NF*κ*B inhibitor, I*κ*B, was lower in SHRSP than in SHR. These results indicate that systemic and local IL-1*β* signals are enhanced in SHRSP compared with WKY or SHR and that this enhancement occurred prior to the onset of stoke.

### 3.3. IL-1*β* Administration Failed to Induce Onset of Stroke, but Increased Incidence of Stroke in SHR

**Figure 3:**
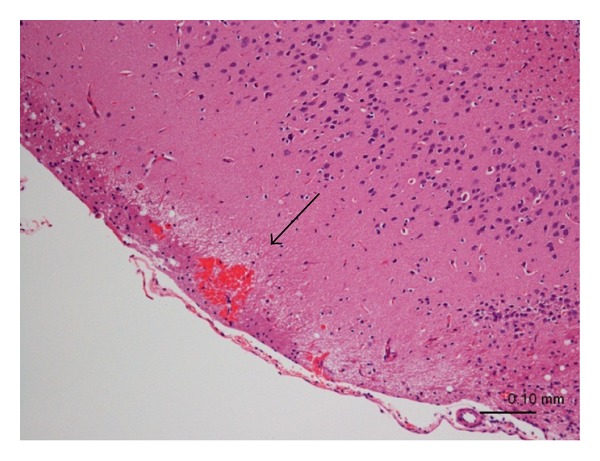
Effect of continuous administration of IL-1*β* on the incidence of stroke in SHR. SHR (male, 10 weeks of age) were given IL-1*β* (2 *μ*g/day) or vehicle (phosphate-buffered saline) for 4 weeks using osmotic pumps. Photograph of typical hemorrhage lesion in SHR given IL-1*β* for 4 weeks.

A primary cause of stroke in SHRSP is severe hypertension; however, it is believed that other factors must also contribute to the onset of stroke in SHRSP. Enhanced IL-1*β* signal in SHRSP might be one of the factors contributing to stroke onset. To clarify this possibility, SHR were given continuous administration of IL-1*β* (2 *μ*g/day) using osmotic pump. The administration of IL-1*β* significantly decreased food intake at 1 and 2 weeks and body weight at 1 week, but did not affect blood pressure compared with control during the 4-week administration period (Table 2). Plasma level of IL-1*β* was significantly increased to almost the same level of age-matched SHRSP within the first week, and this increase persisted for at least 3 weeks with IL-1*β* administration (Table 2). However, none of the animals in either group showed signs of stroke (neurologic symptoms and physiological changes) during the 4-week administration period, possibly because blood pressure was still lower in SHR than SHRSP (Tables 1 and 2). To determine the effect of IL-1*β* on the incidence of stroke, histological analysis was conducted. Histological analysis showed that IL-1*β* administration slightly increased the incidence of stroke in SHR, even though each lesion was small and did not induce any sighs of stroke onset (Table 3 and Figure 3). These results suggest that IL-1*β* might be a cause of stroke onset, but it could not exacerbate stroke lesions without the presence of severe hypertension.

### 3.4. IL-1*β* Administration Accelerated the Onset of Stroke in SHRSP

To address whether IL-1*β* signal plays a role in stroke onset in SHRSP with severe hypertension, SHRSP underwent continuous administration of IL-1*β* (2 *μ*g/day) using an osmotic pump for 4 weeks. The administration of IL-1*β* significantly decreased food intake and slightly decreased body weight, but did not affect blood pressure compared with control at 1 week after administration (Table 4). The first animal in the IL-1*β* group showed stroke sign at day 9. Thus, we stopped to measure blood pressure and withdraw blood to avoid any influences on the onset of stroke. Plasma level of IL-1*β* was significantly higher in the IL-1*β* group than in control. In this condition, the administration of IL-1*β* significantly accelerated the onset of stroke compared with control (*P* = 0.006) (Figure 3(a)), even though administration of IL-1*β* did not affect blood pressure (Table 4). There was a significant inverse correlation between plasma IL-1*β* level and the day of stroke onset in all rats (*r* = −0.600 [*P* = 0.007]) (Figure 3(b)). However, the number of rats was limited, and additional experiments are needed to ensure the inverse correlation between plasma IL-1*β* level and the day of stroke onset. Moreover, the administration of IL-1*β* also shortened lifespan compared with control although this difference did not reach statistical significance (Figure 3(c)). To address the effect of IL-1*β* on the exacerbation of stroke lesion and stroke types, autopsy analysis was conducted. There were no differences in severity and incidence of infarction, hemorrhage, and edema between the control and IL-1*β* groups (Table 5). These results suggest that IL-1*β* signal can trigger stroke onset in SHRSP but not exacerbate each type of stroke lesion.

## 4. Discussion

Although signs of inflammation are present within stroke lesions, it is unclear whether inflammation is a cause or a result of stroke. Inflammatory conditions, such as IL-1*β* level in plasma and IL-1*β* signal-related gene expressions in CVECs, are greater in SHRSP compared with WKY or SHR prior to the onset of stroke. In addition to these physiological conditions in SHRSP, IL-1*β* administration slightly increased the incidence of stroke in SHR and significantly accelerated the onset of stroke in SHRSP. These data suggest that inflammation, especially IL-1*β* signal, might play an important role in stroke onset.

Clinical studies using statins already use inflammatory events as endpoints for stroke prevention. A meta-analysis of statin trials showed that statins might reduce the incidence of all strokes by decreasing low-density lipoprotein-cholesterol without increasing the incidence of hemorrhagic stroke [17]. In addition to cholesterol-dependent effects, cholesterol-independent effects of statins on stroke have also been recognized [18, 19]. However, statin treatment increased the risk of hemorrhagic stroke in patients with a history of cerebrovascular disease, even though it also clearly decreased the risk of ischemic stroke [20]. Therefore, patients undergoing statin treatment should be carefully monitored to avoid achieving very low level of cholesterol, which is a well-known risk factor for hemorrhagic stroke [21].

IL-1*β* may be a more important target for prevention or treatment of stroke when compared with IL-6 and TNF-*α*. Plasma levels of IL-6 and TNF-*α* were similarly low in all rat strains in the present study. In addition, IL-6 mediates anti-inflammatory effects in addition to its proinflammatory role [22], and therefore, its manipulation can have either detrimental or beneficial effects. A previous report also suggests that TNF-*α* has a dual role in brain injury [23]. Although anti-TNF-*α* strategies have proved beneficial in other clinical settings such as inflammatory bowel disease, there have been no clinical trials of anti-TNF-*α* agents in stroke. In contrast, IL-1 is uniquely placed as a therapeutic target of stroke. Chronic increase in IL-1*β* expression in the brain led to leukocyte infiltration and increased MCP-1 and ICAM-1 expressions in a mouse model [24], which is a phenotype also seen in stroke lesions. In addition, a number of studies have demonstrated that inhibiting the release or actions of IL-1 markedly reduces ischemic cerebral damage. IL-1*α* and IL-1*β* double knockout mice exhibited dramatically reduced ischemic infarct volume compared with wild-type mice [25]. A meta-analysis of animal studies showed that IL-1 receptor antagonist (IL-1Ra), which is the most advanced approach to modify IL-1 actions, reduced infarct volume in animal models of focal cerebral ischemia [26]. In humans, a phase II clinical trial of intravenous IL-1Ra compared with placebo in patients with acute stroke is currently under way [27]. Further, IL-1Ra gene polymorphisms represent a risk factor for ischemic stroke [28]. These reports suggest that the inhibition of IL-1*β* signal might prevent or delay the onset of stroke.

We tried to inhibit IL-1*β* signal using anti-rat IL-1*β* polyclonal antibody. The administration of anti-IL-1*β* antibody (600 *μ*g/day) slightly, but not significantly, delayed the onset of stroke compared with control (*P* = 0.232 by the Log-Rank test). There are some reasons that anti-IL-1*β* antibody did not delay the onset of stroke. First, anti-rat IL-1*β* polyclonal antibody did not work *in vivo*. Plasma level of IL-1*β* (74.0 ± 14.7 pg/mL) did not change by anti-IL-1*β* antibody administration after 1 week. It seems that anti-IL-1*β* antibody failed to inhibit IL-1*β* signal, including IL-1*β*-positive feedback regulation. However, anti- IL-1*β* antibody could suppress the IL-1*β*-induced COX-2 mRNA expression in SHRSP CVECs *in vitro* (data not shown), suggesting that the anti-IL-1*β* antibody did not work under *in vivo* conditions. Second, doses of anti-IL-1*β* antibody used in this study may not have been large enough to delay stroke onset. The Administration of anti-IL-1*β* antibody slightly affected the onset of stroke, but it seems that higher doses may be needed to see a greater effect. Unfortunately, more concentrated antibody levels would not work using the osmotic pump. We should address this issue in another study.

IL-1R type I and IL-1R type II mRNA levels in CVECs were higher in SHRSP than in WKY or SHR. IL-1R type I is a signal receptor, whereas IL-1R type II is traditionally known as a decoy receptor that dampens the inflammatory response. Recently, it was reported that IL-1R type II mediated internalization and/or transport of IL-1*β* through the blood brain barrier [29]. It is not clear whether IL-1R type II has a proinflammatory or anti-inflammatory effect in SHRSP CVECs, but expression of downstream genes, such as ICAM-1 and MCP-1, was significantly higher in CVECs from SHRSP than WKY or SHR. These results suggest that the IL-1*β* signal was enhanced in SHRSP CVECs, even if IL-1RII acts as a decoy. In addition, NF*κ*B is a key molecule involved in stroke [30], and p50 and RelB proteins in CVECs were greater in SHRSP than in WKY or SHR. Our data support observations of increased NF*κ*B expression in stroke lesions in humans [31].

The hypertensive phenotype may also contribute to the stimulated IL-1*β* signal in SHRSP, because hypertension causes endothelial dysfunction and induces vascular inflammation [32]. CVECs used in our experiments were harvested from 4 weeks of age, which is when blood pressure in SHRSP did not differ from SHR; however, enhanced IL-1*β* signal in CVECs was observed in SHRSP compared with SHR. Thus, the stimulated IL-1*β* signal might be established genetically and independently on hypertension. Moreover, age-related increase in blood pressure could exaggerate vascular inflammation in SHRSP. However, causes of stroke are more complicated in humans and are related to a variety of factors such as hypertension, obesity, diabetes, dyslipidemia, oxidative stress, infection, and inflammation [33]. More studies are needed to characterize the role of the IL-1*β* signal in stroke onset in humans.

## 5. Conclusions

Hypertension is a predominant risk factor of stroke in SHRSP and in humans. Our study suggests that inflammation, especially IL-1*β* signal, might be a risk for stroke onset. More studies are needed to characterize the role of the IL-1*β* signal in stroke onset, and to confirm if this signal might be a therapeutic target for stroke in humans.

## Figures and Tables

**Figure 4:**
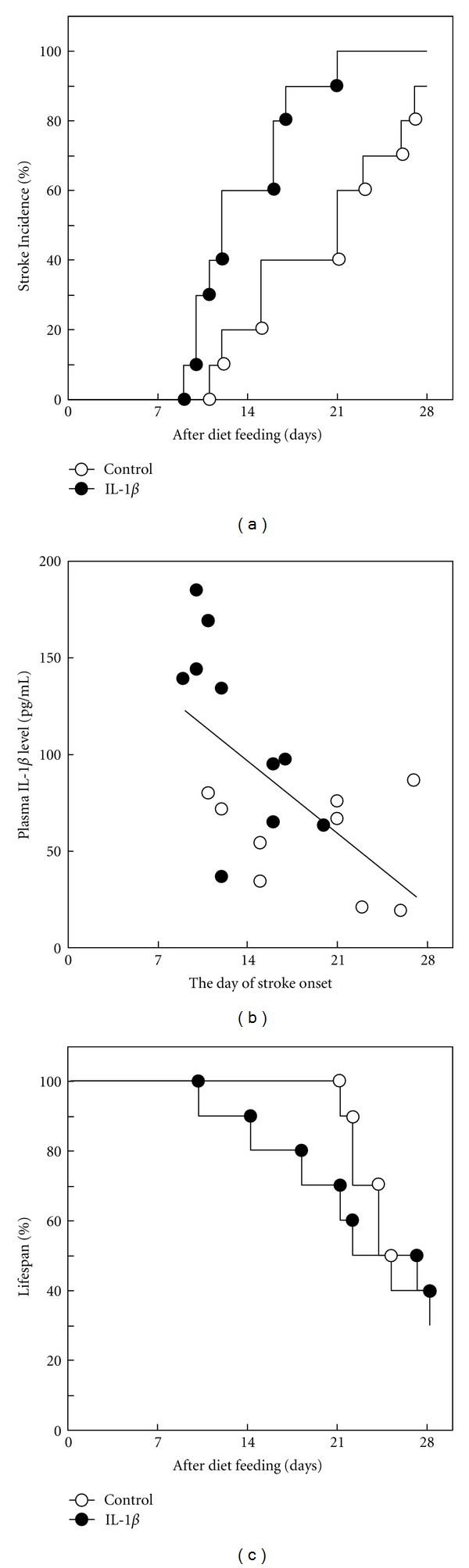
Effect of continuous administration of IL-1*β* on the onset of stroke in SHRSP. SHRSP (male, 10 weeks of age) were given IL-1*β* (2 *μ*g/day) or vehicle (phosphate-buffered saline) for 4 weeks using osmotic pumps. Day of stroke onset was determined as described in the methods. (a) Incidence of stroke. *n* = 10 in each group. (b) Correlation between plasma IL-1*β* level (measured at 1 week after administration; before any animals did not show stroke sign) and the day of stroke onset. Open circles, control (*n* = 9; one rat did not show any stroke sign until 4 weeks); closed circles, IL-1*β* (*n* = 10). (c) Lifespan during the 4-week administration period.

**Table 1:** Body weight, tissue weight, and blood pressure of each strain.

	Age	WKY	SHR	SHRSP	*P* value
Body weight (g)	4	117.1 ± 6.8	123.4 ± 7.8	104.7 ± 4.6^∗††^	0.002
	10	266.0 ± 10.9	237.9 ± 6.1***	221.0 ± 6.8^∗∗∗††^	<0.001
Liver (g)	4	5.33 ± 0.40	5.72 ± 0.45	4.28 ± 0.08^∗∗∗†††^	<0.001
	10	8.12 ± 0.36	8.00 ± 0.22	7.47 ± 0.19^∗∗∗††^	0.002
Vis-Fat (g)	4	0.30 ± 0.06	0.32 ± 0.03	0.27 ± 0.04	0.236
	10	3.19 ± 0.30	2.46 ± 0.33**	2.00 ± 0.33^∗∗∗†^	<0.001
SBP (mmHg)	4	107 ± 8	138 ± 12***	146 ± 7***	<0.001
	10	122 ± 7	165 ± 7***	190 ± 9^∗∗∗†††^	<0.001

Data presented as mean ± SD. Body weight and weights of liver, epididymal adipose tissue (Vis-Fat), and systolic blood pressure (SBP) were measured at 4 and 10 weeks of age after overnight fasting. A *P* value of one-way ANOVA is shown. *P < 0.05, **P < 0.01, ***P < 0.001 versus WKY, ^†^ *P* < 0.05, ^††^ *P* < 0.01, ^†††^ *P* < 0.001 versus SHR. *n* = 5 at 4 weeks of age or 6 at 10 weeks of age in each strain.

**Table 2:** Effect of continuous IL-1*β* administration to SHR.

		Before	1 week	2 weeks	3 weeks	4 weeks
BW (g)	Control	268.7 ± 9.5	295.5 ± 10.4	316.0 ± 11.7	327.9 ± 10.4	335.4 ± 10.7
	IL-1*β*	266.4 ± 8.8	290.3 ± 11.2	300.0 ± 10.1**	317.9 ± 11.2	326.8 ± 11.4
SBP (mmHg)	Control	173 ± 11	158 ± 12	181 ± 17	173 ± 14	181 ± 8
	IL-1*β*	175 ± 10	161 ± 11	172 ± 16	171 ± 10	174 ± 14
IL-1*β* (pg/mL)	Control	24.6 ± 15.5	26.1 ± 5.9	33.5 ± 17.7	37.9 ± 12.6	21.9 ± 8.2
	IL-1*β*	25.4 ± 19.2	65.4 ± 18.8***	66.5 ± 20.6***	61.2 ± 30.7*	24.3 ± 6.2
Intake (g/day)	Control		23.9 ± 1.1	20.8 ± 1.1	20.6 ± 0.7	18.7 ± 0.9
	IL-1*β*		22.6 ± 1.1*	19.7 ± 1.0*	20.8 ± 0.9	19.0 ± 0.9

Data presented as mean ± SD. Body weight (BW), systolic blood pressure (SBP), and plasma level of IL-1*β* were measured at before (10 weeks of age) and every one week of IL-1*β* administration. Food intake (Intake) is presented as the mean weight of food eaten per day during each week. *P < 0.05, **P < 0.01, ***P < 0.001 versus Control by unpaired Student *t*-test. *n* = 10 in each group.

**Table 3:** Histological analysis of the brain in SHR.

	Infarction	Hemorrhage	Edema
Control	1	5	0
IL-1*β*	4	10	2

After 4 weeks of IL-1*β* administration, rats were killed and histological analysis was conducted. Incidence of infarction lesion, hemorrhage lesion, and edema in 6 sections of each rat, and 10 rats of each group were counted. *P* = 0.046 versus Control by Fisher's exact probability test.

**Table 4:** Effect of continuous IL-1*β* administration to SHRSP.

		Before	1 week
BW (g)	Control	249.3 ± 7.0	276.7 ± 6.2
	IL-1*β*	248.4 ± 13.5	263.2 ± 21.0
SBP (mmHg)	Control	202 ± 20	203 ± 16
	IL-1*β*	200 ± 14	199 ± 16
IL-1*β* (pg/mL)	Control	62.5 ± 29.2	56.1 ± 24.5
	IL-1*β*	62.6 ± 31.8	112.2 ± 49.0*
Intake (g/day)	Control		23.2 ± 0.5
	IL-1*β*		17.9 ± 1.1***

Data presented as mean ± SD. Body weight (BW), systolic blood pressure (SBP), and plasma level of IL-1*β* were measured before (10 weeks of age) and 1 week after the initiation of IL-1*β* administration. Food intake (Intake) is presented as the mean weight of food eaten per day during the first week of IL-1*β* administration. *P < 0.05, ***P < 0.001 versus Control. *n* = 10 in each group.

**Table 5:** Magnitude and location of infarction, hemorrhage, or edema for individual rats in each group from Figure 4.

Location	Infarction						Hemorrhage						Edema					
	1	2	3	4	5	6	1	2	3	4	5	6	1	2	3	4	5	6
Control																		
Rat #1	−	−	++	−	−	−	−	−	+	−	−	−	−	+++	++++	++	−	−
2	−	−	−	−	−	−	−	−	−	−	−	−	−	−	−	−	−	−
3	++	−	+++	++++	−	−	++	−	+++	++++	−	−	++	++	+++	++++	−	−
4	−	−	−	−	−	−	−	−	−	−	−	−	−	−	−	−	−	−
5	−	−	−	−	−	−	−	−	−	−	−	−	+	++	−	−	−	−
6	−	−	−	−	−	−	−	−	−	−	−	−	−	−	−	−	−	−
7	−	−	−	−	−	−	−	−	+	−	−	−	−	−	−	−	−	−
8	+	++	+++	−	++++	−	+	++	+++	−	−	−	−	++	+++	++	++++	−
9	+	+++	−	−	−	−	−	+++	−	−	−	−	++++	++	−	−	−	−
10	−	−	−	−	−	−	−	−	−	−	−	−	+++	++	−	−	−	−

IL-1*β*																		
Rat #1	+	++	−	−	−	−	+	++	−	++	−	−	+	++	−	+	−	−
2	−	−	−	−	−	−	−	−	++	++	−	+	−	−	−	−	−	−
3	−	++	−	−	−	−	−	+	−	+	−	−	+	++	++	++	−	−
4	−	+++	+++	−	−	−	−	++	++	++	−	−	+++	+++	++	++	−	−
5	−	−	−	−	−	−	−	−	−	−	−	−	−	−	−	−	−	−
6	++++	−	−	−	−	−	++	−	−	−	−	−	++++	+	−	−	−	−
7	−	−	+	+	−	−	−	−	−	−	−	−	−	−	−	−	−	−
8	+	−	+	++++	++	−	−	−	++	++++	++	−	+++	+++	++	++	−	−
9	+++	++	−	+	−	−	+++	−	−	+	−	−	++	+	+	+++	−	−
10	−	−	+++	−	+++++	−	−	−	−	−	−	−	−	−	−	++++	+++++	−

The brain was removed after death and fixed with 10% buffered formalin solution. Locations 1–6 indicate the section of stroke: 1 is frontal, 6 is rear. Increasing number of + indicates stronger changes, − indicates no abnormality. Rats were numbered in order of the onset of stroke; #1 was the first rat where stroke occurred in each group.
